# Rapid genome sequencing for pediatrics

**DOI:** 10.1002/humu.24466

**Published:** 2022-09-23

**Authors:** Jana Jezkova, Sophie Shaw, Nicola V. Taverner, Hywel J. Williams

**Affiliations:** ^1^ All Wales Medical Genomics Service, Cardiff and Vale NHS Trust Heath Hospital Cardiff UK; ^2^ Centre for Medical Education, School of Medicine Cardiff University Heath Park Cardiff UK; ^3^ Division of Cancer and Genetics, Genetic and Genomic Medicine, School of Medicine Cardiff University Heath Park Cardiff UK

**Keywords:** bioinformatics, ethics, next‐generation sequencing (NGS), rare disease, variant interpretation, whole exome sequencing (WES), whole genome sequencing (WGS)

## Abstract

The advancements made in next‐generation sequencing (NGS) technology over the past two decades have transformed our understanding of genetic variation in humans and had a profound impact on our ability to diagnose patients with rare genetic diseases. In this review, we discuss the recently developed application of rapid NGS techniques, used to diagnose pediatric patients with suspected rare diseases who are critically ill. We highlight the challenges associated with performing such clinical diagnostics tests in terms of the laboratory infrastructure, bioinformatic analysis pipelines, and the ethical considerations that need to be addressed. We end by looking at what future developments in this field may look like and how they can be used to augment the genetic data to further improve the diagnostic rates for these high‐priority patients.

## 1 BACKGROUND

In the years following the publication of the first draft of the Human Genome Project (Lander et al., 2001; Venter et al., 2001) technological advances have vastly improved our ability to analyze the genome. This has resulted in an increasing shift from single gene testing using the costly and time‐consuming Sanger sequencing technique to next‐generation sequencing (NGS)‐based multigene testing. NGS was initially used for academic research but soon thereafter, it began to be translated into the clinic. Today, the use of NGS within the clinical setting has become routine for the diagnosis of patients with rare diseases (RD) and cancer.

Although the cost of NGS has fallen dramatically over the last decade, driven by tremendous advancements in technology, the cost of whole exome sequencing (WES) and whole genome sequencing (WGS) (collectively referred to as genomic sequencing here on in) is still a barrier to many diagnostic laboratories and it is therefore pertinent to use it where it has the highest likelihood of identifying a disease‐causing mutation and can, therefore, have the biggest impact on patient well‐being.

There are estimated to be around 10,000 individual RDs (Haendel et al., 2020) which collectively affect hundreds of millions of patients worldwide. However, it is thought that up to 80% of these diseases have a genetic component, which means that elucidation of the molecular cause of the disease is amenable to NGS. Finding the molecular cause of a disorder gives us vital insights into the pathobiology of these diseases, which in turn improves our understanding of the biological pathways affected and offers hope for the development of novel therapeutics.

To maximize the limited funds available to perform clinical NGS diagnostics, it is necessary to use the available resources in the most efficient and cost‐effective way. This is by no means straightforward as multiple factors need to be considered, which will be unique for each setting. For example, the use of unbiased genomic sequencing instead of disease‐specific gene panels or single gene tests avoids the need to perform multiple sequential tests if the first one comes back negative. This is particularly useful because every time a new causative gene is identified for an RD, gene panels need to be updated to incorporate it at much time and expense. The downside to WGS and, to a lesser extent WES, is their increased sequencing costs and the extra bioinformatic burden associated with analyzing and storing the huge amounts of data generated with these techniques. Nonetheless, WGS can be thought of as a form of investment because once you have the data from the whole genome, it can be used to retrospectively investigate any novel findings that may be published after the initial analysis has been performed. It may also be cost‐effective to target patients with RDs that have been shown to be highly tractable to genomic sequencing approaches, such as those with a neurodevelopmental phenotype, in which a diagnostic rate of up to 70% can be obtained (Acuna‐Hidalgo et al., 2016; Brunet et al., 2021; Deciphering Developmental Disorders Study, 2017; Heyne et al., 2018; Kaplanis et al., 2020; Pode‐Shakked et al., 2021; Samocha et al., 2014).

For this review, we will focus on the burgeoning field of rapid diagnosis of critically ill pediatric RD patients who are in paediatric and neonatal intensive care units (PICU and NICU). For this unique cohort of patients, there are many clinical benefits to receiving a time‐critical clinical diagnosis and many cost benefits for the healthcare provider. First, because the patients are young and not yet fully developed, it is far more difficult for clinicians to make an accurate diagnosis based on their phenotype, meaning a genetic test can be the best way of reaching a confirmatory diagnosis. Also, an early diagnosis provides knowledge to inform clinical management on the best therapeutics to use, which can reduce the time to treatment and improve outcomes. There are also financial benefits to decreasing the number of costly days in the intensive care unit for neonates or children (NICU/PICU) (Farnaes et al., 2018; Lunke et al, 2020; Sanford Kobayashi et al., 2021; Stark, Boughtwood, et al, 2019; Yeung et al., 2020).

The vital term here is “rapidly” because the health benefit for the patient and cost‐effectiveness the healthcare provider can achieve is determined by the speed to which a diagnosis can be made. The first study to demonstrate the feasibility of performing rapid WGS (rWGS) in a PICU setting was published in 2012 by Saunders and colleagues, who showed it was possible to reach a diagnosis in just 50 h (Saunders et al., 2012). In comparison, it typically takes 1–6 months following NGS testing to arrive at a diagnosis in most clinical settings.

Since this time, more than 20 studies have been published from around the world describing the use of rapid genomic sequencing in over 1500 patients, representing a range of healthcare settings (reviewed in [Stark & Ellard, 2021]). Two notable randomized clinical trials, NSIGHT1 (Petrikin et al., 2018) and NICUSeq (Krantz et al., 2021) have shown that rWGS can be implemented into routine clinical practice and leads to a change of the clinical management of critically ill children.

There is now unprecedented evidence to show the clinical utility of this approach and the economic healthcare advantages it offers (see also articles in this series) (Goranitis et al., 2022). The advances in this field have been made through technical improvements of the sequencing instruments, the use of improved bioinformatic hardware/software, and through an alignment of the disparate experts who come together in such a healthcare setting to deliver the best care possible for their patients. In fact, these advances have resulted in a new world record time of 5 h 2 min for the fastest DNA sequencing technique to sequence an entire human genome and the shortest time from sample receipt to diagnosis of 7 h 18 min (Gorzynski et al., 2022).

## 2 CURRENT STATE OF PLAY IN RAPID GENOME SEQUENCING

The maturity of rapid genomic sequencing in a critical care setting is such that its translation and implementation into routine clinical practice has been successfully achieved in a growing number of countries such as the United Kingdom, Australia, and the United States. In the United Kingdom, funding for most genomic tests, including rapid genome sequencing, is government‐based and is provided at the national level within the National Health Service (NHS). The NHS in England has implemented rWES for critically ill children since October 2019. This test is for acutely unwell children with a likely monogenic disorder when a diagnosis is required more urgently to aid clinical management, prenatal testing, or pre‐implantation genetic diagnosis. Of 361 children enrolled during the first year, 141 (38%) received a diagnosis. In 133 (94%) patients, the molecular diagnosis influenced clinical management (Stark & Ellard, 2021).

The NHS in Wales is the first service in the United Kingdom to introduce a national diagnostic rWGS service for critically ill newborns and children as a front‐line test. In 2019, the All Wales Medical Genomics Service formed a multidisciplinary working group tasked with designing and implementing this service. New diagnostic testing infrastructure was established and a bespoke diagnostic pipeline to identify causative genetic variants was validated. The “Wales Infants' and childreN's Genome Service” (WINGS) was launched in April 2020. Patients are eligible for the service if a monogenic cause for their illness is suspected, a DNA sample from both biological parents is available, and a timely genetic diagnosis might alter clinical management. The service is available to pediatric and neonatal patients in intensive care units (ICUs) across Wales, and Welsh children in ICUs elsewhere in the United Kingdom (Murch et al., 2021). The test can be ordered by a NICU or PICU consultant or registrar (equivalent to specialist and trainee) following a telephone discussion with the on‐call clinical genetics team.

Forty‐five families have completed testing in the first 2 years of the WINGS service. Pathogenic or likely pathogenic variants have been identified in 17 children. Additionally, in two cases, variants of uncertain significance (VUS) have been reported. Approval to report VUS that are relevant to patient's phenotype and incidental findings must be sought from multidisciplinary teams. These are teams of clinical scientists and consultants from clinical genetics, pediatrics, biochemistry, and other specialties that are involved in the patient's care and who meet ad hoc to discuss more complex genomic results.

Mean time to reporting was 9 calendar days (range 3–26 days). These results have had significant health benefits for this patient group, including immediate clinical management changes. The highest diagnostic yields were identified in children with either neurological (57%) or metabolic (60%) phenotypes (where *n* > 4 patients) (personnel communication). The overall diagnostic yield of 37.5% is similar to previous research projects and other services internationally (French et al., 2019; Kingsmore et al., 2019; Mestek‐Boukhibar et al., 2018).

Elsewhere, a pilot quality improvement study “Project Baby Bear” run in California, became the first state‐funded program to use rWGS as a first‐line diagnostic test for critically ill newborns with suspected rare genetic diseases in the United States (Dimmock et al., 2021). Led by Rady Children's Institute for Genomic Medicine, the study provided rWGS for 178 infants enrolled in California's Medicaid program (MediCal) hospitalized in intensive care with an aim to evaluate the clinical benefits and economic impact of this test. The data, collected in 18 months, showed that rWGS resulted in a diagnostic yield of 43%. The findings led to a change in care in 31% of the solved cases while saving $2.5 million in healthcare costs. Based on the success of Project Baby Bear, the “Ending the Diagnostic Odyssey Act 2021” was introduced which allows all 50 state Medicaid programs to cover rWGS for eligible individuals (Collins, 2021).

In 2016, The Australian Genomics Health Alliance (Australian Genomics) was launched as a national collaborative research partnership of more than 80 organizations. Its aim was to integrate genomics as the standard of care into the Australian healthcare system using a whole‐of‐system approach, building the evidence to inform national health policy (Stark, Schofield, et al., 2019).

The Australian Genomics Acute Care program built upon the prior experience of implementing rWES across two hospitals in 2016–2017. Participants were acutely unwell pediatric inpatients (0–18 years) with suspected monogenic disorders. The study provided a diagnosis for 52.5% patients, changed management of 57% diagnosed patients, and showed that diagnosis by rWES costs half that of diagnosis by usual care (Stark et al., 2018). A more recent scaled‐up study investigated the feasibility of ultra‐rWES in critically ill pediatric patients with suspected monogenic conditions in the Australian public healthcare system. This multisite study, which included 12 hospitals and 2 laboratories, aimed to deliver genomic results within 5 days to 108 patients. Similarly to the previous study, NICU or PICU patients with a likely monogenic disorder were eligible if they had been referred to the clinical genetics service. Other inpatients were also included if a rapid result was likely to alter clinical management (e.g., organ transplant decisions). The diagnostic yield was 51% and the mean time to report was 3.3 days (Best et al., 2021; Lunke et al, 2020). In July 2020, the study team received further funding to drive the expansion of this service and transition to WGS.

These examples highlight the astonishing progress made in the field of pediatric rapid‐diagnostics and the translation of it from a research endeavor to a routine clinical test. However, implementing a test such as rapid genomic sequencing in a clinical setting still poses a number of challenges (described below) that need to be overcome before it can be adopted more widely.

## 3 CHALLENGES SURROUNDING RAPID GENOMIC SEQUENCING AND BIOINFORMATICS

For rapid genome sequencing to be clinically useful and financially effective, it is imperative that all steps along the workflow are optimized to run smoothly and efficiently. After sample collection, there are certain steps that are difficult to speed up, for example, it takes a set time to extract DNA from blood. Some steps are already optimized, such as the commonly used sequencing library preparation kits purchased from commercial vendors, and other steps can be streamlined using automation, such as liquid handling robots. It is noteworthy to highlight that if rWES is being performed, then the hybridization stage will result in a longer library preparation time compared to rWGS (~2 days for trio rWES vs. ~2.5–3 h for trio rWGS). In all cases, an optimized and well‐communicated sample triage, testing, and analysis workflow is crucial to the efficient processing of the sample through diagnostics, improving turnaround times for patients.

Access to an appropriate NGS platform is again essential to the timely processing of the sample, as well as being able to produce sufficient depth of coverage in a cost‐effective manner. In general, a depth of coverage of at least 20× across the genome is required to accurately identify single nucleotide changes. Illumina sequencing machines are commonly used by clinical laboratories and researchers as a standard device, however, several models are available, with differing specifications. For human WGS, the NovaSeq system is recommended, with four flow cells available for use, all with differing capabilities. This ranges from between four and 48 human genomes in a single run, taking between 25 and 44 h, producing up to 3000 Gb of data. Table 1 lists the differing specifications for 100 bp paired‐end reads, but specifications differ again depending on the choice of read length. Therefore, careful planning and management are needed to ensure that the correct flow cells and settings are being used in each case. In addition, advancements in long‐read sequencing technology have also been recently used to demonstrate the use of long reads in rWGS (Goenka et al., 2022).

**Table 1:** Illumina NovaSeq. 6000 flow cell specifications for 2 × 100 bp paired end reads^a^

Flow cell type	SP	S1	S2	S4
Number of human genomes per run	~4	~8	~20	~48
Output per run	135–167 Gb	266–333 Gb	667–833 Gb	1600–2000 Gb
Run time	~19 h	~19 h	~25 h	~36 h

^a^ Information taken from https://emea.illumina.com/systems/sequencing-platforms/novaseq/specifications.html. Specifications for v1.5 reagent kits as of September 2022.

**Figure 1:**
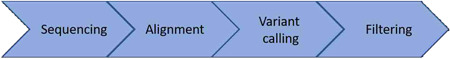
Typical bioinformatics whole genome sequencing analysis pipeline. After sequencing, fastq files are produced. These are aligned to the reference genome to produce alignment files, typically in BAM (binary alignment) format. These are used for variant calling to produce VCF (variant call format) files, which are then annotated and filtered to produce the final variant list.

The output from a genomic sequencing run is a set of fastq files that contain the sequence data for the millions/billions of bases of DNA along with quality score metrics. To take this data and convert it to manageable information on genetic variation, efficient, accurate, and validated bioinformatics analysis pipelines are needed. All pipelines follow the same key steps from quality filtering, then alignment to the reference genome, followed by variant calling, and finally variant annotation (Figure 1). These analyses can be computationally intensive and time‐consuming, performing complex tasks such as implementing algorithms to align millions of reads to the three billion base pair human reference genome. Due to this complexity, it is unsurprising that processing of a single genome can take ~36 h, even on a large well‐powered compute cluster (Goranitis et al., 2022). Choice of software appropriate for the analysis task is key to both accuracy and run time of the pipeline, with a large number of studies published comparing software options (Chen et al., 2019; Hatem et al., 2013; Kumaran et al., 2019; Musich et al., 2021). Attempts to standardize these approaches have been made, with the best practice guidelines recommended by the Broad Institute for use with their Genome Analysis ToolKit (GATK4), which is commonly used, although there are other available options (Van der Auwera & O'Connor, 2020). A recent review (Koboldt, 2020) discusses further options for best practices for clinical variant calling including software choices for aligners and variant callers. In terms of maintaining the accuracy of the pipeline, but in a rapid manner, recent innovations have been made including the development of BWA‐MEM2, a faster version of the popular Burrow–Wheeler Aligner (BWA) software (Vasimuddin et al., 2019), the closed source DRAGEN™ Bio‐IT Platform (https://emea.illumina.com/products/by-type/informatics-products/dragen-bio-it-platform.html) (Illumina) which encompasses all stages of analysis allowing a trio of whole genomes to be processed in around 6 h, and the open source Dragmap version of the DRAGEN aligner (https://github.com/Illumina/DRAGMAP) (Illumina), making this computing capability available to all.

As with the wet laboratory work, options are available to optimize these bioinformatics processes, such as the utilization of high‐performance compute clusters with a batch‐queuing system, allowing for parallelization of tasks; the use of sophisticated workflow languages, such as nextflow (https://www.nextflow.io/) and snakemake (Molder et al., 2021); and simple solutions such as networking the sequencers to allow for direct saving of the data to the compute cluster, removing the need for lengthy transfer of raw data, which can also lead to corruption or loss of data.

Before any sample going through a bioinformatics pipeline for diagnosis, substantial groundwork is needed to validate the process to ensure accuracy. This encompasses use of knowns, such as genome in bottle samples (Zook et al., 2016), and in‐house previously identified samples from separate platforms, to calculate the specificity and sensitivity of the pipelines. Care must also be taken to ensure that all potential sample types can be used, that processing is efficient, and that the pipeline is producing usable outputs for clinical scientists. ACGS have published guidelines for best practices in the validation of bioinformatics pipelines (Whiffin et al., 2016) and Marshall and colleagues (2020) have recently published a review on best practices for validation.

**Figure 2:**
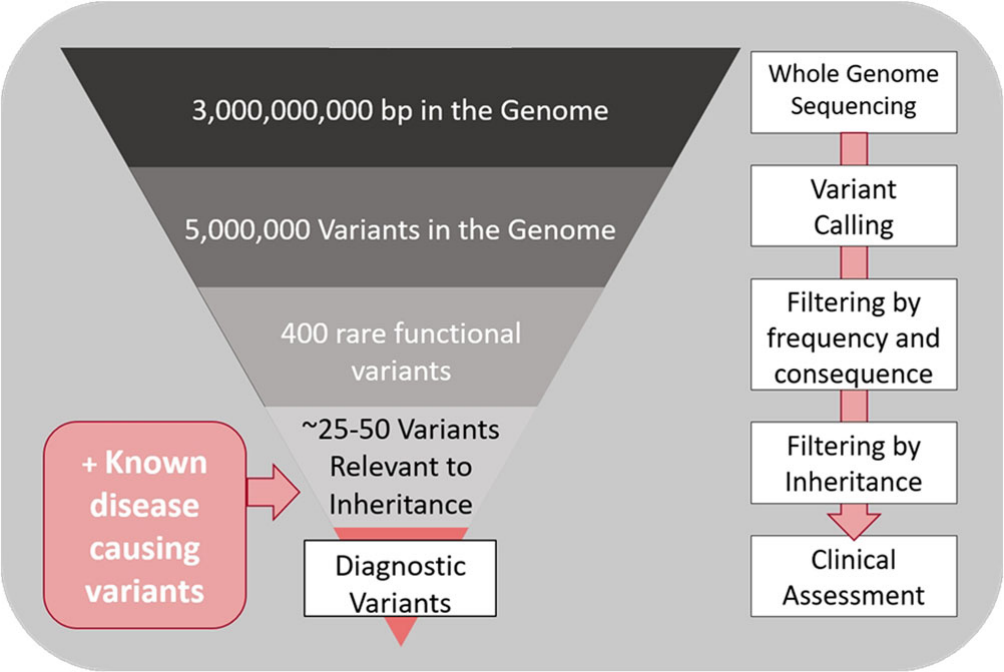
Example filtering strategy for genome sequencing analysis

Once the variant data is in the form of a vcf file (variant call format) it next needs to be annotated with functional information such as variant consequence, the frequency of the variant in the population (Karczewski et al., 2020), and a range of other metrics that assess the potential of the variant to be pathogenic (Adzhubei et al., 2010; Kumar et al., 2009; Lek et al., 2016; Rentzsch et al., 2019; Williams et al., 2022). This annotation step is carried out by specialist software, such as Variant Effect Predictor (McLaren et al., 2016), which searches a series of predownloaded databases for this information. Armed with this information and the patient's phenotypic information, a diagnosis is made by a clinical geneticist/clinical scientist according to set guidelines (Richards et al., 2015) agreed to by the clinical diagnostic community. However, without an appropriate filtering strategy (Figure 2), the number of variants could be as high as several million, a completely unmanageable number for assessment.

Filtering strategies include applying hard cut offs based on metrics such as base quality, mapping quality, and coverage; removal of noncoding variants; filtering by variant consequence; filtering by prevalence in the population using GnomAD; and filtering by inheritance pattern where trios are available (Wright et al., 2018). The biggest challenge is to narrow down the list of variants to a manageable amount, ensuring rapid analysis by the clinical scientists, but while also ensuring that any potential causative variants are not removed. With appropriate filtering, clinical scientists can be left with around 25–50 variants to manually assess.

This filtering can be aided by the addition of “white lists” of known clinical, pathogenic variants, such as the use of ClinVar variants. This strategy was suggested in the PAGE study looking at pre‐natal rWES for fetal diagnostics (Lord et al., 2019). In addition, a “gene panel” approach is often applied, focusing only on those genes associated with the phenotype or condition. These gene panels are readily available from resources such as the Genomics England PanelApp (Martin et al., 2019) and are regularly reviewed and updated, however, they do restrict the analysis to known disease‐associated genes and can, therefore, miss variants in genes with novel associations. A similar approach is taken to removal of noncoding variants, unless they are already known to have a clinical impact, again risking missing novel pathogenic noncoding variants. Reaching a diagnosis requires an understanding of how the implicated gene might impact the patient's phenotype, as well as how the variant identified might affect the function of the protein, requiring extensive biological knowledge by clinical scientists.

The assignment of a diagnosis can be another time‐consuming step as each variant passing the filtering criteria needs to be interpreted individually. To speed up this process, progress has been made in the use of automated machine‐learning methods that combine the patient's phenotypic information with the details from the diagnostic guidelines, this approach has been shown to result in a time saving of ~22 h (Clark et al., 2019). However, this is still an active area of research and is not widely implemented.

**Figure 3:**
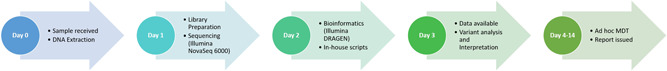
Laboratory pathway for Wales Infants' and ChildreN's Genome Service (WINGS). Rapid whole genome sequencing (WGS) is available for acutely unwell children with a likely underlying genetic cause. Genomic DNA extracted from the child's and parent's blood samples undergoes genomic sequencing using the Illumina NovaSeq. 6000 system. Sequences are aligned to human genome assembly GRCh38 (hg38), and variants identified with the Illumina DRAGEN (Dynamic Read Analysis for GENomics) Bio‐IT Platform (v.3.7; Illumina). Analysis includes evaluation of variants that are identified to be de novo, compound heterozygous, homozygous, and X‐linked using in‐house bioinformatic pipelines. Variants are interpreted and reported following the latest ACGS/American College of Medical Genetics and Genomics (ACMG) guidelines (Ellard et al., 2020; Richards et al., 2015). Only causative pathogenic and likely pathogenic variants are reported, variants of uncertain significance (VUS) that are potentially related to the child's illness are discussed at a multidisciplinary meeting and may also be reported. The reporting time for this test is 14 calendar days.

With the addition of some of these time‐saving capabilities, a sample can go from receipt at the diagnostic center, to a potentially classified variant in just 3 days (Figure 3).

In summary, sample preparation, sequencing, and bioinformatics remain challenging area in rapid whole genome diagnostics. Careful planning and thorough validation are required to ensure that all stages within the sample pathway are accurate and optimized.

## 4 ETHICAL AND INCIDENTAL FINDINGS CHALLENGES

Alongside the technical challenges of implementing rapid genomic sequencing, there are also ethical and practical challenges to offering such services. Ethical issues can include obtaining informed consent, the discovery of incidental findings unrelated to the reason for testing, the privacy of genomic data, the possibility of discrimination based on the findings, the potential impact on the parent‐child relationship, and the prioritization of resources in a publicly funded health service. Stark and Ellard (2021) discussed these ethical challenges in their recent review, acknowledging that many of these issues are common to any genomic sequencing, but may be compounded by the clinical situations in which rapid genomic sequencing is being offered, when individuals or their carers are being asked to make decisions about genomic testing quickly and when they or their relatives are in vulnerable situations. This makes it harder to achieve informed consent, though Stark and Ellard's review found that most parents consenting for rapid genome sequencing for their unwell newborn child did not regret testing and believed it to be useful. Generally, the parents' focus was on diagnosis and rapid genome sequencing provided this opportunity, though they did report some challenges associated with consenting for this testing and many felt overwhelmed. Similarly, healthcare professionals viewed rapid genomic sequencing as being very helpful for clinical diagnosis, though generally felt that this should be led by the genetics team who have greater knowledge about genomic testing as well as expertise in providing information and support to individuals making these decisions and adapting to the results (Stark & Ellard, 2021).

One key issue is the possibility of identifying incidental or additional findings unrelated to the symptoms under investigation, which indicate that the individual and potentially their family members are at risk of other health conditions. It can be argued that this is a benefit for medically actionable conditions, as appropriate management can be put in place to reduce the risk or achieve better outcomes for the individual, and the American College of Medical Genetics and Genomics (ACMG) has published a list of conditions for which incidental findings from genomic screening should be reported (Miller et al., 2021). It is worth noting that this list is not definitive and will change over time with increases in our understanding and changes in the treatment and management options available, so individuals tested at different times may not be tested for the same conditions. For some conditions, it has been argued that testing should go even further, with the possibility of systematically screening children for familial hypercholesterolemia (an inherited form of high cholesterol that can lead to heart attacks and strokes) to identify their parents who may be at risk (Wald & Wald, 2019). However, incidental findings may also relate to conditions that are not medically actionable (i.e., there is no screening or management available to improve health outcomes), in which case the balance of benefits and harms in reporting these findings is more debatable. With Huntington's disease, a degenerative condition, only around a fifth of those with a 50% chance of having the causative genetic variation chose to have presymptomatic testing to find out whether or not they would develop the condition in later life (Baig et al., 2016). Therefore, though many think that they would like to know predictive information about their future health (Middleton et al., 2015), when individuals are faced with finding out this kind of information about the future, many preferred not to know. Those being offered genomic testing to try to identify a diagnosis for their seriously unwell relative are unlikely to think carefully about whether or not they would want to know this kind of incidental information.

The chance of identifying incidental findings is influenced by the filtering strategy used as part of the pathway, as discussed above. While the whole genome is sequenced, the data analysis can be adapted as desired. For example, a gene panel approach can be used, only looking at genes known to be associated with a genetic disease or even only those genes associated with a particular phenotype. However, it could be argued that this misses an opportunity to identify medically actionable genetic conditions (such as those on the ACMG list), leaving individuals unaware of their risk, with the resulting impact on health outcomes in later life and on healthcare costs. It would also be important to ensure that patients and their healthcare professionals are aware that, while the genome has been sequenced, it has not all been analyzed, so some genomic variants will have been excluded. Even if testing covered genes associated with genetic disease as well as these medically actionable conditions, this testing strategy relies on current genomics knowledge and means that novel causes of genetic conditions will not be identified, reducing diagnostic yield. Therefore, a gene agnostic approach may be preferable, identifying potentially pathogenic variants in all parts of the genome, with the associated risk of incidental findings. A slightly modified version could be considered, excluding particular genes associated with disease which is not medically actionable to maintain a higher diagnostic yield but reduce the chance of these findings. However, again, it may be difficult to reach consensus as to which genes should be excluded. If they are to be excluded, it may be more practical to do this at the data analysis stage, rather than carrying out a full analysis and not reporting these findings. However, patients and their families may start to request their raw genomic data for analysis using various online services, so these incidental findings may be identified elsewhere.

Implementation of rapid genomic testing pathways needs to include consideration of who will be tested, what will be tested, and the associated clinical pathway. As outlined above in the discussion of the current state of play, testing is offered to those who are acutely ill with a likely monogenic disorder where testing is likely to make a difference to management. In addition, DNA samples have been required from both parents to enable analysis, which has implications for equality of access, as this excludes some patients from testing if both parents are not available. However, as the technology moves beyond the pilot stage into routine practice and our knowledge and analysis improve, it becomes increasingly possible that trio analysis will not be essential.

Clinical judgment is required to target testing appropriately to these patients, and both time and expertise are needed to provide this service, which has implications for workforce planning so services looking to implement rWGS will need to consider how this can be managed. As with many specialties, it may be necessary both to obtain expertise from other hospitals or areas, as well as upskilling local staff to meet the needs of patients. Genomic testing should be offered to patients by healthcare professionals, such as genetic counselors, with both a good understanding of genomic testing and also the skills to help individuals with decision‐making. This will facilitate the provision of informed consent for testing, though it could be argued that it is not possible to obtain fully informed consent due to the breadth of possible findings that can arise. These staffs need to be well informed about the testing being offered, the potential findings that could be obtained and also what may not be revealed by testing. They also need to have the skills to deliver the results, and provide support to help individuals and families assimilate and adapt to their results.

If incidental findings are discovered in infants and children, the parents will be given this information and it will be important to consider how this will be provided to the child themselves as they grow older, to avoid a further ethical issue of others knowing about a risk of which the individual themselves is unaware. Again, healthcare professionals giving the results should support parents with considering how and when the information will be passed to the child, and may need to work with families to ensure that they have the skills, knowledge, and intention of passing this information to the individual as they become older.

As highlighted by this discussion, it is important for healthcare professionals offering WGS to have a good understanding of what testing is being carried out, what may be found and missed, and the ethical issues associated with this. Guttmacher et al.'s (2007) review notes a range of studies indicating a lack of genomics knowledge and confidence in nongenetics medical professionals from a range of countries and specialties. Therefore, there is a need for genomics education both of medical and other healthcare professionals. In the United Kingdom, genomics education has been incorporated into medical school curricula and, within England, a national approach to genomic education for health professionals is being coordinated by Health Education England's Genomic Education Programme (Slade et al., 2016). However, it will take time to upskill all healthcare professionals to provide genomic testing across the UK's National Health Service.

## 5 FUTURE RAPID‐TESTING STRATEGIES

Finally, it is worth looking to the future to think about what additional rapid tests could be translated to augment the analysis of the genomic data. The reason for this is based on the fact that while the application of rapid genomic sequencing has greatly facilitated the identification of disease‐associated genetic variants in critically ill children, around two‐thirds/half of patients remain undiagnosed. This is partly due to challenges in interpretation of genomic variants and our limited understanding of how variants impact gene expression and protein abundance, as well as protein structure and interactions but also due to our failure to identify some types of disease‐causing variants, such as deep intronic variants, noncoding triplet repeats, variants in enhancer and promoter regions, and larger structural variants.

When rapid genomic sequencing returns inconclusive results, analyzing multiple layers of biological activity together can help us better understand the functional aspects of genomic variants and their role in disease. The functional genomics techniques that can be utilized for this purpose are transcriptomics, epigenomics, proteomics, or metabolomics. For example, metabolic and biochemical tests can guide genomic analysis or provide insights in the pathogenicity of variants within genes involved in metabolic pathways and are already routinely used to aid clinical diagnosis.

For the purpose of this review, we will focus on the use of transcriptomics as a complimentary test to genomic sequencing, as there is already mounting evidence to show its potential utility. Studying the transcriptome, RNA expressed from the genome, provides valuable pathogenicity information on sequence variants. RNA studies can validate candidate splice‐disrupting mutations, confirm whether candidate truncating variants cause nonsense‐mediated decay, and identify splice‐altering variants in both exonic and deep intronic regions.

The main limitation of transcriptome studies is an accessible tissue source with suitable expression levels of clinically relevant genes. For patients, this often means additional invasive sampling such as a skin biopsy followed by establishing fibroblast cell line cultures. Fibroblasts express the majority of known disease genes (Yépez et al., 2022) and can be used to derive pluripotent stem cells which express over 27,000 genes (Bonder et al., 2021). In a rapid setting culturing might not be possible due to time constraints, therefore, blood sampling might be preferred. Multiple studies support the use of blood as a viable source of RNA material in different RD including neurological disorders. To help overcome the limitation of tissue specificity, resources are now available that help identify clinically accessible tissues (i.e., MAJIQ‐CAT [Aicher et al., 2020] and asses the feasibility of RNA‐sequencing, i.e., MRSD [Rowlands et al., 2021]).

Initially, targeted studies using reverse transcription polymerase chain reaction (RT‐PCR) have been utilized to functionally validate and reclassify VUSe for some time now (Le Quesne Stabej et al., 2017; Wai et al., 2020). More recently, the reduced cost of RNA sequencing (RNAseq) make it an equally viable option not only for confirmation of candidate variants, but also to perform a transcriptome‐guided genomic analysis. The use of transcriptomics as a secondary diagnostic tool has been extensively reviewed elsewhere. Briefly, transcriptome‐wide RNA‐seq data can be used to streamline and to direct downstream analysis by prioritization of causative variants that have been overlooked or completely filtered out by genomic sequencing. RNAseq has been shown to identify pathogenic variants that have been missed by DNA‐based testing alone, improving diagnostic yield by 7.5%–36% across a diverse range of rare disorders (Cummings et al., 2017; Frésard et al, 2019; Gonorazky et al., 2019; Lee et al., 2020; Maddirevula et al., 2020; Murdock et al., 2021; Rentas et al., 2020; Yépez et al., 2021, 2022)

One notable study (Murdock et al., 2021) used a novel transcriptome‐directed analysis approach to provide diagnoses for patients with rare Mendelian disorders. Instead of looking at candidate genes derived from DNA sequencing, the authors suggest starting with RNAseq to direct the prioritization of DNA variants. The study demonstrates the clinical application of the Detection of RNA Outlier Pipeline (DROP) (Yépez et al., 2021), an automated workflow that detects genes with aberrant expression, aberrant splicing, and mono‐allelic expression of genes in whole blood and fibroblasts. This approach resulted in a diagnostic yield of 17% in patients with a wide range of conditions including neurological, musculoskeletal, and immune phenotypes to detect aberrant expression and splicing.

## 6 CONCLUSIONS

The evolution of genomic sequencing since the completion of the Human Genome Project has transformed our understanding of how human genetic variation can lead to RD's. Within the clinical setting, NGS techniques are routinely used to diagnose RD patients, with the recent 100,000 Genomes Project demonstrating a diagnostic rate of 25% in patients spanning a wide‐spectrum of clinical phenotypes (Smedley et al., 2021).

Nonetheless, there are still barriers to implementing genomic sequencing for clinical diagnostics that include costs, availability of trained personnel, and the huge bioinformatic/compute infrastructure required to process, interpret and store patient's genomic data in a safe environment. It is thus necessary to identify areas where the implementation of genomic sequencing can have a large positive impact.

We argue that, given the evidence described above, the use of rapid genomic sequencing to diagnose acutely ill children with a suspected monogenic disease is such an environment. There is compelling evidence to show that being able to rapidly diagnose such children can lead to improvements in clinical management. The rapid nature of the tests also leads to substantial healthcare cost reductions for the healthcare provider as the children can be treated quicker and moved to less high‐dependency beds.

In the future, we believe rapid genomic sequencing will become common practice for healthcare providers across the globe, and advances in technology will improve the time to diagnosis as well as costs. Orthogonal techniques such as RNAseq will augment the genomic data and undoubtedly improve diagnostic rates even further. There is, therefore, much anticipation to see how this exciting field will evolve and the promise it holds to improve the diagnosis for critically ill children.

## CONFLICT OF INTEREST

The authors declare no conflict of interest.

## Data Availability

There is no data to make available as this review did not generate any data.
